# Analysis of volatile compounds in *Aglaia odorata* flower extracts with different possessing methods by HS-SPME-GC-MS and E-nose

**DOI:** 10.3389/fchem.2026.1746408

**Published:** 2026-03-06

**Authors:** Pengfei Yang, Lingqi Kong, Qiongbo Wang, Qiang Liu, Xiujin Duan, Chen Hu, Zhengbo Feng, Qi Yang, Huabo Jv

**Affiliations:** 1 College of Tobacco Science and Engineering, Zhengzhou University of Light Industry, Zhengzhou, Henan, China; 2 School of Public Health and Nutrition, Luohe Medical College, Luohe, Henan, China; 3 Technology Center, China Tobacco Henan Industrial Co., Ltd., Zhengzhou, Henan, China; 4 Gansu Tobacco Quality Supervision & Test Station, China National Tobacco Corporation, Lanzhou, Gansu, China

**Keywords:** *Aglaia odorata* flower extracts, headspace solid phase microextraction-gas chromatography-mass spectrometry, multivariate statistical analysis, relative odor activity value, volatile components

## Abstract

**Introduction:**

To investigate the impact of different processing methods on the volatile components in *Aglaia odorata* flower extract (AOFEs).

**Methods:**

Headspace solid-phase microextraction coupled with gas chromatography-mass spectrometry and electronic nose (E-Nose) analysis were employed to characterize volatiles of extracts obtained by ultrasound-assisted extraction (UAE), microwave-assisted extraction (MAE), and heated reflux extraction (HRE). Multidimensional assessment using aroma radar charts, orthogonal partial least squares-discriminant analysis (OPLS-DA), K-means clustering, and relative odor activity value (ROAV) revealed significant processing-dependent variations.

**Results and discussion:**

The results indicated that the fiber coated with DVB/CAR/PDMS had optimal extraction efficiency. A total of 46 compounds were identified, including eight alcohols, four aldehydes, one acid, 25 terpenes, seven ketones, and one heterocyclic compound. UAE and MAE had 36 and 38 compounds respectively, sharing similar compositional profiles but differing in concentrations, while HRE produced only 25 compounds Sensory evaluation and E-Nose results revealed differences in the aroma profiles of the extracts, with UAE and MAE extracts exhibiting intensified floral and sweet notes, whereas HRE displayed prominent green and spicy characteristics. K-means clustering categorized volatile evolution trends into four distinct subclasses. OPLS-DA identified 13 differential volatiles with variable importance in projection greater than 1, with ROAV analysis further selecting eight key markers, including (1R,7*S*)-germacra-4(15),5,10(14)-trien-1*β*-ol, *α*-humulene, copaene, *β*-cadinene, (E)-*β*-caryophylene, *Γ*-cadinene, humulene oxide II, and caryophyllene oxide. These compounds collectively contribute to the sweet and floral attributes of AOFEs. This study elucidated extraction-method-dependent volatile profiles and aroma characteristics, providing theoretical guidance for process optimization and quality enhancement in AOFEs production.

## Graphical Abstract

**Figure:**
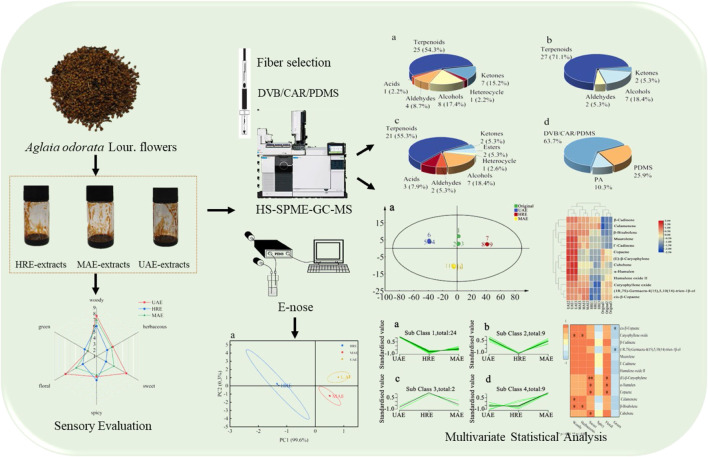

## 1 Introduction

*Aglaia* *odorata*, a member of the *Aglaia genus* within the Meliaceae family, is a woody plant predominantly inhabiting sparse forests and shrublands in low-altitude mountainous regions (Hao et al., 2024; Yin et al., 2024). Endemic to southeastern Asia, its natural distribution spans southern China, Vietnam, India, Thailand, and Malaysia; in China specifically, it is primarily cultivated in Fujian and Guangdong provinces, with additional wild or cultivated populations present in Guangxi, Yunnan, and Sichuan (Hao et al., 2024). The defining characteristic of *Aglaia odorata* lies in its tiny, grain-like flowers that emit an intense, long-lasting fragrance—a unique blend of jasmine, ylang-ylang, and tea notes that underpins their significant economic value in the fragrance and cosmetics industries. It has been used as a traditional herb to treat heart disease, bruises, traumatic injury, and pyresis (Hisashi et al., 2016). Furthermore, derived natural fragrance products such as essential oils, absolutes, tinctures, and concretes (*Aglaia odorata* flower extracts, AOFEs) have gained widespread application in food flavoring, cosmetics manufacturing, soap production, and tobacco flavoring, owing to their superior olfactory quality and favorable physiological activities (Yang et al., 2022). Among these products, concretes have attracted particular attention due to its advantages of simple processing technology and high yield. Ultrasound-assisted extraction (UAE), microwave-assisted extraction (MAE), and heated reflux extraction (HRE) are common processing techniques for concrete preparation (Chan et al., 2016; Chemat et al., 2017; Hoang et al., 2025). Relevant studies have shown that there are significant differences in the volatile components of plant concretes obtained by different processing methods (Cao et al., 2024; Khan et al., 2025). As important quality markers of natural fragrances, differences in volatile components can lead to variations in the quality of AOFEs, thereby affecting their applications. However, few studies have investigated the differences in volatile substances among AOFEs produced by different preparation methods. Therefore, it is essential to conduct a comparative analysis of the differences in volatile components of AOFEs prepared using different processing methods to clarify the impact of processing methods on these volatile components.

Headspace solid-phase microextraction (HS-SPME) is a solvent-free extraction technique for volatile components (Lancioni et al., 2022). By selecting different extraction fiber coatings, this technique effectively enriches trace volatile compounds, offering advantages such as high selectivity, ease of operation, and rapid processing (Liang et al., 2023). It integrates sampling, extraction, concentration, and injection into a single process. It has been widely applied in plants and foods (Shuai et al., 2024; Song et al., 2021). Gas chromatography-mass spectrometry (GC-MS) is an effective method for detecting and analyzing volatiles; it is usually combined with HS-SPME and extensively used in the detection and analysis of volatile compounds in various foods, such as alcoholic beverages (Milheiro et al., 2023), fruits (Song et al., 2021; Zhang et al., 2019), tea (Du et al., 2024), etc. HS-SPME combined with GC-MS (HS-SPME-GC-MS) enables rapid detection and analysis of volatile components in AOFEs, but it cannot conduct intuitive sensory evaluation. In contrast, electronic nose (E-nose) technology based on sensory analysis identifies flavor components by using different aroma sensors to mimic human olfactory functions (Yue et al., 2024). Hence, combining HS-SPME-GC-MS with E-nose can better distinguish the differences in volatile components and sensory characteristics of AOFEs prepared by different processing methods. While instrumental measurements offer objective data on product characteristics, they do not necessarily correlate with hedonic experiences; therefore, sensory evaluation remains indispensable for validating the practical significance of experimental findings.

Based on this, the study aims to analyze and identify volatiles of the AOFEs prepared by different methods (UAE, MAE and HRE) via HS-SPME-GC-MS combined with E-nose technology and to determine the key differential components in the AOFEs obtained from each method by multivariate statistical analysis. The result of this study can provide theoretical guidance and scientific basis for the process optimization and quality improvement of AOFEs.

## 2 Materials and methods

### 2.1 Chemical and reagents

Borneol (97%), *β*-guainene (90%), *δ*-cadinene (95%), sulcatone (98%), limona ketone (98%), camphor (99%), hendecane (99%), dodecane (99.5%) and tridecane (99%) were purchased from Adamas Reagent Co., Ltd., Shanghai, China. (*Z*)-3-Hexenol (99%), (*S*)-verbenone (95%), (*E*)-2-hexenal (98%), heptanal (97%), 3-butylacrolein (95%), benzenecarbonal (99%), nonanal (98%), decanal (97%), (-)-camphene (95%), caryophyllene oxide (95%), 1-methylpyrrole (99%), 2-pentylfuran (98%), ethyl octoate (99%) and ethyl pelargonate (95%) were purchased from Aladdin Reagents Co., Ltd., Shanghai, China. 3-Ethyl-4-methylpentanol (98%), *β*-safranal (95%), junipen (95%) were obtained from Accela ChemBio Co., Ltd., Shanghai. Spatulenol (93%), (-)-palustrol (98%), (1*R*,7*S*)-germacra-4(15),5,10(14)-trien-1*β*-ol (97%), longicyclene (98%), (*E*)-*β*-Caryophylene (98%), *α*-humulene (93%) and humulene oxide II (97%) were gotten from yuanye Bio-Technology Co., Ltd., Shanghai, China. 2,6-Dimethylundecane (95%) was obtained from TCI Development Co., Ltd., Shanghai, China. Copaene and *β*-selinene were gained from Toronto Research Chemicals. Psoraderm (98%), *α*-curcumene (98%) and viridflorol (98%) were purchased from BioBioPha Co., Ltd. C_6_-C_30_ n-alkanes, ethanol (HPLC grade) and ethyl decanoate (99%) were gotten from J&K Chemicals Ltd., Beijing, China. Sodium chloride (analytical grade) was obtained from Sinopharm Chemical Reagent Co., Ltd.

### 2.2 Plant materials and AOFEs preparation

#### 2.2.1 Plant materials

*Aglaia odorata* flowers (AOFs) were purchased in Zhangzhou City, Fujian Province, in 2024 and authenticated by a botanist prior to use. The flowers were crushed and passed through an 80-mesh sieve for later use. The powder of AOFs was used as the original sample for later use.

#### 2.2.2 Preparation of AOFEs samples

UAE-extracts: Weigh 10.0 g of AOF powder into a round-bottomed flask, adding 100 mL of 80% (v/v) ethanol solution, and performed ultrasonic extraction at 60 and 300 W for 30 min (KQ-500DE CNC Ultrasonic Cleaner, Kunshan Ultrasonic Instrument Co., Ltd.). The UAE-extracts was obtained by removing the solvent from the extract using a rotary evaporator at 30 °C and 40 mbar for 10 min.

HRE-extracts: Weigh 10.0 g of AOF powder into a round-bottomed flask, adding 100 mL of 80% (v/v) ethanol solution, and then heated at 80 °C for 4 h (HH-S2 Digital Display Constant Temperature Water Bath, Jiangsu Jinyi Instrument Technology Co., Ltd.). Removed the solvent from the extract using a rotary evaporator at 30 °C and 40 mbar for 10 min to obtain the HRE-extracts.

MAE-extracts: Weigh 10.0 g of AOF powder into a round-bottomed flask, adding 100 mL of 80% (v/v) ethanol solution and conduct a microwave extraction at 240 W for 20 min (WBFY-205 Microwave Chemical Reactor, Gongyi Yuhua Instrument Co., Ltd.). Removed the solvent from the extract using a rotary evaporator at 30 °C and 40 mbar for 10 min to obtain the MAE-extracts.

All the above samples were prepared in triplicate and keep in 4 °C until analysis.

### 2.3 E-nose

A PEN3.5 portable E-nose (Airsense, Germany) was used to detected the odor of AOFEs. Accurately weigh 1.0 g AOFEs and placed into a 20 mL headspace vial, heating at 60 °C for 10 min, then equilibrated at room temperature for 10 min. The headspace gas was taken for detection, and each sample was tested in triplicate. The parameters of the E-nose were set as follows: sample detection time of 120 s, sensor cleaning time of 300 s, automatic zero adjustment time of 10 s, and injection flow rate of 400 mL/min. The average value of the response values from 117 s to 119 s of each sample was used for subsequent analysis (Han et al., 2025). The sensitive substances of the 10 metal detectors in the electronic nose were shown in Table 1.

**TABLE 1:** Ten sensors used in E-nose and their substances for sensing.

Nos.	Sensor	Sensibility
1	W1C	Aromatic components
2	W5S	Nitrogen oxides, broad range
3	W3C	Ammonia and aromatic components
4	W6S	Mainly hydrogen, selectively, (breath gases)
5	W5C	Alkanes and aromatic components
6	W1S	Propane, broad range
7	W1W	Sulfur organic compounds
8	W2S	Ethanol
9	W2W	Aromatic components and organic-sulfides
10	W3S	Propane (selective sometimes)

### 2.4 HS-SPME

Accurately weigh 1.0 g sample into a 20 mL headspace vial, then added 2 mL saturated NaCl aqueous solution and 50 μL of internal standard solution (ethyl decanoate, 0.78 mg/mL in absolute ethanol). Sealed the vial with a cap equipped with a polytetrafluoroethylene septum, and placed it in an automatic HS-SPME device. After heating and equilibrating at 60 °C for 20 min, pushing the activated extraction probe into the headspace vial for extraction lasting 20 min. Upon completion of extraction, the automatic extraction device inserted the extraction probe into the GC-MS injection port, where desorption was performed at 270 °C for 4 min (Pati et al., 2021).

The fiber coating of the extraction probe can selectively adsorb volatile components, so it is necessary to screen fiber probes with different polarities (Mebazaa et al., 2009). The types of probes investigated in this experiment were shown in Table 2. HS-SPME was performed on 1.0 g original sample (the powder of AOFs) using probes with fiber coatings of different polarities. Combined with GC-MS, the types and contents of volatile compounds extracted by the three fiber coating probes were analyzed to select the optimal fiber probe for subsequent experiments. All experiments were repeated three times.

**TABLE 2:** Fibers used in HS-SPME.

Coating material	Abbreviation	Film thickness	Length	Aging conditions
Polyacrylate	PA	85 μm	1 cm	270 °C, 1 h
Polydimethylsiloxane	PDMS	100 μm	1 cm	250 °C, 0.5 h
Divinylbenzene-carboxen-polydimethylsiloxane	DVB/CAR/PDMS	50/30 μm	1 cm	270 °C, 1 h

### 2.5 GC–MS analysis

A gas chromatography (Agilent 8890B) coupled with an Agilent 5977 mass spectrometer detector (MSD) on DB-WAX capillary column (30 m × 0.25 mm, 0.25 µm) with helium (≥99.999% purity), at a flow rate of 1.0 mL/min, as the carrier gas. A splitless injection mode was employed during the volatile insertion at 270 °C. The oven temperature was raised from 40 °C (held for 4 min) to 125 °C at a rate of 10 °C/min, and then ramped to 180 °C at a rate of 1.5 °C/min. The temperatures of the ion source and transfer line between the GC and MS were set at 230 °C and 280 °C, respectively. The quadrupole temperature was 150 °C and the solvent delay was 6.4 min. Detection and quantification were carried out with electron ionization (70 eV) and the full scan range was 35–500 amu.

### 2.6 Qualitative and quantitative analyses

For the qualitative analysis of volatile compounds, mass spectral data corresponding to each chromatographic peak were extracted from the total ion current (TIC) chromatogram of the volatile components and matched against the NIST20 mass spectral library for identification. Components with a matching degree of over 80% were selected, and further verified by comparing with their retention times with those of reference standards analyzed under the same conditions (Molyneux and Schieberle, 2007).

Volatile compounds were quantified using the internal standard method, with ethyl decanoate as the internal standard. The concentration of each compound in original sample and AOFEs was calculated based on the following formula:

$$W=\frac{S_{0}\times W_{1}}{S_{1}}$$

Where *W* is the volatile concentration, *S*_*0*_ represents the peak area of the volatiles, *W*_*1*_ is the internal standard concentration, *S*_*1*_ represents the peak area of the internal standard (Yang and Cadwallader, 2025).

### 2.7 Relative odor activity value

The relative odor activity value (ROAV) of volatile components in original sample and AOFEs were calculated according to Li et al. (2024). Volatile component that contributes the most to the flavor of the extracts was defined as ROAV_stan_. The formula for calculating the relative ROAV of volatiles was as follows:

$${\text{ROAV}}_{ᵢ}\approx \left(C_{ᵢ}/C_{stan}\right)\times \left(T_{stan}/T_{ᵢ}\right)\times 100$$

Where *Cᵢ* is the concentration of each volatile compound, μg/g; *Tᵢ* is the threshold of each volatile compound, μg/g; *C*_*stan*_ is the concentration of the volatile compound that contributes the most to the overall aroma of the samples, μg/g; *T*_*stan*_ is the threshold of the volatile compound that contributes the most to the overall aroma of the samples, μg/g.

The identification of key aroma compounds depends not only on their concentration levels but also on their ROAV. A higher ROAV indicates a greater contribution of that compound to the overall aroma profile (Guo et al., 2022; Wang et al., 2020). It is generally accepted that when ROAV > 1, the aroma compound plays a dominant role in the overall aroma and can be considered a key aroma compound of the sample; when ROAV > 0.1, the aroma compound has a significant modifying effect on the overall flavor (Yang et al., 2025).

### 2.8 Sensory evaluation

The sensory evaluation was conducted on AOFEs according to the method specified in GB/T 14454.2-2008 (2008). Eleven professionals (six males and five females) who had received prior training for 3 weeks and passed the olfactory test were selected. In the first round of training, the evaluators need to be familiar with the smell of various standards and their corresponding sensory descriptors, and further smell the AOFEs obtained by different extraction methods to be familiar with their aroma characteristics; in the second round of training, the evaluators selected and recorded the descriptors from their respective smell. After the discussion of the team members, six aroma descriptors were finally determined: woody, herbaceous, sweet, spicy, floral and green. In the process of evaluation, A scale from zero to nine was used, where zero indicated no aroma and nine indicated an extremely strong aroma. The average score of the 11 professionals for each aroma attribute was taken as the final aroma intensity.

### 2.9 Statistical analysis

All data are reported as the mean ± standard deviation of three tests. SPSS 27.0 software was used to analyze the data using Duncan’s multiple range tests of variance (*p* < 0.05) and a significance test. Origin 2021 was used to conduct Principal Component Analysis (PCA), bar charts, Venn diagrams, and Pearson correlation heatmaps. The E-nose loadings plot was generated with Winmuster software and the K-means clustering line graph was plotted using R software. Orthogonal partial least squares discriminant analysis (OPLS-DA) and variable importance in projection (VIP) were completed with SIMCA 14.1 software. Hierarchical clustering analysis (HCA) were done using the TB tools.

## 3 Result and discussion

### 3.1 Fiber selection

**FIGURE 1:**
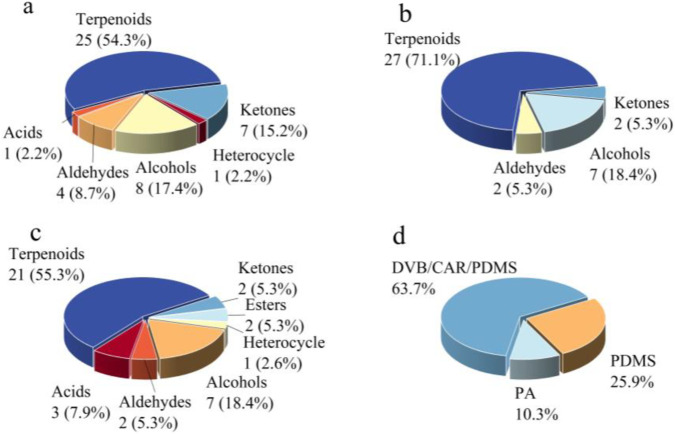
Types of compounds (**(a)** DVB/CAR/PDMS; **(b)** PDMS; **(c)** PA) and their total peak areas **(d)** extracted from different fiber coating.

Properties of the fiber coating are the main factors that determine the extraction efficiency (Kim et al., 2020). Different cladding materials have various polarities, which can selectively adsorb volatiles (Kataoka, 2021). Figure 1 had shown the extraction effects of fiber coatings with different polarities. Among them, the medium-polarity probe (DVB/CAR/PDMS) had detected 46 compounds in total, which consisted of eight alcohols, four aldehydes, one acid, 25 terpenoids, seven ketones, and one heterocyclic compound (Figure 1a). The non-polar probe (PDMS) has also detected 38 compounds, which include seven alcohols, two aldehydes, 27 terpenoids, and two ketones (Figure 1b). The polar probe (PA) had detected a total of 38 compounds, including seven alcohols, two aldehydes, three acids, 21 terpenoids, two ketones, two esters, and one heterocyclic compound (Figure 1c). Terpenoids constitute the largest proportion, accounting for over 50%. In addition, the total content of volatile compounds extracted by the medium-polarity probe (DVB/CAR/PDMS) had been significantly higher than that of the other two probes (Figure 1d). Relevant literature had reported that fiber probes coated with DVB/CAR/PDMS had exhibited high efficiency and reproducibility in extracting flavor compounds with high boiling points (Mebazaa et al., 2009). Therefore, the medium-polarity probe (DVB/CAR/PDMS) has been selected as the optimal extraction fiber probe for volatile compounds.

### 3.2 E-nose

**FIGURE 2:**
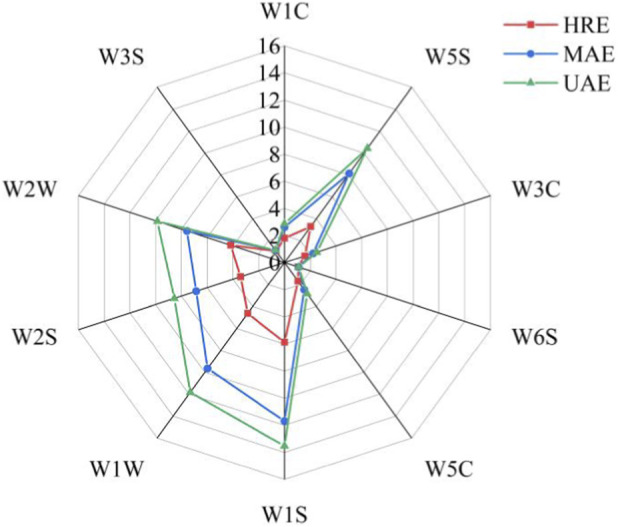
Radar chart of E-nose analysis of AOFEs under different processing methods.

The E-nose sensors are extremely sensitive to volatile components within their detection range. Their response intensity is closely related to the concentration of the corresponding compounds in the sample. Even slight changes in concentration can lead to differences in sensor responses (Fan et al., 2024). As demonstrated in Figure 2, the response values of the ten electronic nose sensors all exhibited differences, yet the overall contour trend remained largely consistent. This finding suggested that there were minor variations in the types of volatile components in AOFEs obtained by the three processing methods. The sensors W5S, W1S, W1W, W2W and W2S exhibited relatively high levels of response intensity, which suggests the presence of abundant volatile components within the AOFEs that were sensitive to these sensors. Furthermore, sensors W1S and W1W demonstrated the highest response values, indicating that the predominant volatile components in the extracts corresponded to terpenoids and alkanes. Previous study had indicated that the volatile compounds of AOFs contained high levels of terpenoids, which was believed to be the key factor contributing to the high response value observed in the W1W sensor (Zhang et al., 2012). The high response value exhibited by the W1S sensor may be attributed to the specific structure of terpenoids present in AOFEs. When terpenoids comprised only carbons and hydrogens with an unsaturation degree of zero, their structural arrangement was analogous to that of alkanes, resulting in a heightened response value for the W1S sensor (Yu and Gu, 2025). A notable finding was the substantial variations observed in the response intensity of E-nose sensors when exposed to AOFEs derived from diverse extraction methodologies. The response value elicited by UAE emerges as the highest, followed by MAE, while HRE elicits the lowest response intensity. The UAE process generated intense shock waves through ultrasonic action, which in turn generated microbubbles that ruptured rapidly. This process enhanced the destruction of cell walls, facilitating the release and dissolution of flavor compounds and thereby producing the most abundant volatile substances (Demesa et al., 2024). MAE and HRE employed soaking and heating methodologies respectively, and had also been shown to effectively extract flavor components. However, their efficiency was marginally lower than that of UAE. HRE primarily relied on steam for heat transfer, which caused the degradation of some thermally volatile components and thus resulted in the lowest content of extracted flavor substances (Xu et al., 2017).

**FIGURE 3:**
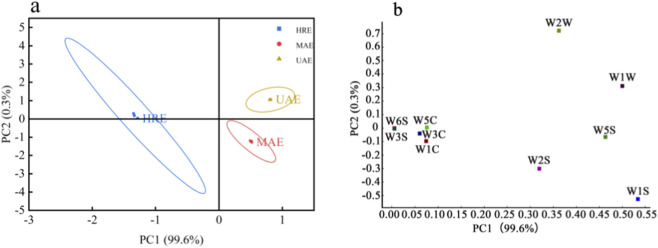
PCA **(a)** and LOA **(b)** plots of AOFEs with different processing methods.

PCA was performed on the E-nose sensor response values of volatile components in AOFEs under the three processing methods, and the results were shown in Figure 3a, with Figure 3b being the corresponding Loadings plot. The cumulative contribution rate of the first and second principal components reached 99.9%, which could effectively represent the odor information of AOFEs (Fan et al., 2024). The PCA plot revealed that HRE was located in the negative PC1, while UAE and MAE were positioned to the positive PC1. In addition, UAE was found in the positive PC2, and MAE was correlated negatively with PC2. The PCA plot clearly demonstrated the separation between the three samples, indicating that the processing methods altered the volatile components of the AOFEs. Load Analysis (LOA) could be used to evaluate the impact of sensors on data. For a specific principal component, sensors with loading parameters close to zero contributed less to the total array response, while sensors with high values were more discriminatory (Su et al., 2014). Figure 3b showed the LOA plot of E-nose sensor response values to AOFEs under different processing methods. In PC1, W1S and W1W had the highest levels of contribution, while in PC2, W2W and W1W exhibited the highest levels of contribution, suggesting that there were substantial variations in terpenoids and aromatic compounds in AOFEs obtained by different extraction methods.

### 3.3 Analysis of volatile compounds in AOFEs

**FIGURE 4:**
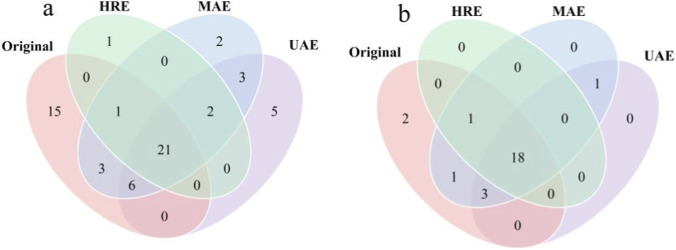
Venn diagrams of total compound species **(a)** and terpenes species **(b)** in AOFEs.

The volatile compounds of AOFEs and original sample were analyzed using HS-SPME-GC-MS. As shown in Table 3, there were significant differences in volatile components between the original sample, UAE-extracts, HRE-extracts, and MAE-extracts. A total of 59 volatile components were detected, which were classified into eight categories, including nine alcohols, seven aldehydes, one acid, 26 terpenoids, seven ketones, five alkanes, two heterocyclic compounds, and two esters. Specifically, the original sample, UAE-extracts, MAE-extracts and HRE-extracts contained 46, 36, 38 and 25 volatiles, respectively. Out of the 59 compounds, 21 were common to all samples (Figure 4a). In addition, five compounds ((*E*)-2-hexenal, undecane, 2,6-dimethylundecane, 5-ethyl-5-methyldecane, and 2-pentylfuran) were only detected in the UAE extract; two compounds ((*E*)-2-heptenal and decanal) were unique to the MAE-extracts; and one compound ((-)-palustrol) was exclusively found in the HRE-extracts. This reflected that UAE had a better extraction effect on specific volatile compounds in AOFs.

**TABLE 3:** Composition and relative content of volatiles in AOF with different processing methods.

Compound	CAS	RI^a^	NIST-RI	Concentration (μg/g)^b^				Identification^c^
				Original	UAE	HRE	MAE	
(*Z*)-3-Hexenol	928-96-1	871	875	0.91 ± 0.19a	-^d^	-	-	MS, RI, S
3-Ethyl-4-methylpentanol	38514-13-5	1,029	1,023	0.19 ± 0.04a	-	-	-	MS, RI, S
(*S*)-Verbenone	1196-01-6	1,162	1,153	0.13 ± 0.04a	-	-	-	MS, RI, S
Borneol	507-70-0	1,194	1,193	0.25 ± 0.00a	-	-	-	MS, RI, S
Spatulenol	6750-60-3	1,562	1,564	5.29 ± 0.14b	23.05 ± 0.14c	3.19 ± 0.52d	11.55 ± 0.37a	MS, RI, S
Viridflorol	552-02-3	1,615	1,612	2.62 ± 0.64b	4.40 ± 0.24b	-	3.28 ± 0.84a	MS, RI, S
(-)-Palustrol	5986-49-2	1,616	1,612	-	-	1.45 ± 0.01a	-	MS, RI, S
(-)-Cubenol	21284-22-0	1,636	1,640	1.11 ± 0.10b	-	-	2.32 ± 0.20a	MS, RI
(1*R*,7*S*)-Germacra-4(15),5,10(14)-trien-1*β*-ol	81968-62-9	1,685	1,694	46.08 ± 2.41b	68.96 ± 1.92c	15.31 ± 0.08d	72.34 ± 0.13a	MS, RI, S
***Alcohols (9)***	​	​	​	**56.57**	**96.41**	**19.96**	**89.49**	​
(*E*)-2-Hexenal	6728-26-3	866	867	-	0.95 ± 0.03a	-	-	MS, RI, S
Heptanal	111-71-7	909	907	0.2 ± 0.01a	-	-	-	MS, RI, S
3-Butylacrolein	18829-55-5	966	964	-	-	-	0.28 ± 0.00a	MS, RI, S
Benzenecarbonal	100-52-7	981	980	0.59 ± 0.23a	-	-	-	MS, RI, S
Nonanal	124-19-6	1,109	1,109	0.47 ± 0.09a	0.57 ± 0.01a	-	0.55 ± 0.04a	MS, RI, S
Decanal	112-31-2	1,211	1,212	-	-	-	0.18 ± 0.02a	MS, RI, S
*β*-Safranal	116-26-7	1,215	1,212	0.14 ± 0.01a	-	-	-	MS, RI, S
***Aldehydes (7)***	​	​	​	**1.41**	**1.52**	**0.00**	**1.01**	​
Caproic acid	142-62-1	990	990	0.73 ± 0.02a	0.9 ± 0.04b	-	0.76 ± 0.00a	MS, RI, S
***Acid (1)***	​	​	​	**0.73**	**0.90**	**0.00**	**0.76**	​
(-)-camphene	79-92-5	961	961	0.14 ± 0.03a	-	-	-	MS, RI, S
Cubebene	17699-14-8	1,354	1,354	35.05 ± 4.98b	68.83 ± 3.68a	30.36 ± 4.35c	37.58 ± 0.16b	MS, RI
*α*-Longipinene	5989-08-2	1,364	1,368	0.23 ± 0.04b	0.56 ± 0.01a	-	0.17 ± 0.02c	MS, RI
Junipen	475-20-7	1,367	1,380	0.41 ± 0.04b	1.66 ± 0.04a	-	0.28 ± 0.09c	MS, RI, S
Cyclosativene	22469-52-9	1,371	1,370	4.65 ± 0.94a	5.4 ± 0.62a	-	1.88 ± 0.21b	MS, RI
Copaene	3856-25-5	1,386	1,387	171.27 ± 22.93d	307.28 ± 3.17b	115.08 ± 0.64a	135.45 ± 8.83c	MS, RI, S
Longicyclene	1,137-12-8	1,426	1,427	0.83 ± 0.19b	1.72 ± 0.02a	0.46 ± 0.07c	0.80 ± 0.13b	MS, RI, S
cis-*β*-Copaene	18252-44-3	1,433	1,433	20.68 ± 1.45b	33.51 ± 1.93a	7.47 ± 0.19c	34.88 ± 8.42a	MS, RI
Psoraderm	484-20-8	1,437	1,435	23.95 ± 2.99d	45.78 ± 0.09a	18.15 ± 0.11c	28.41 ± 1.09b	MS, RI, S
*β*-Guainene	88-84-6	1,445	1,447	2.34 ± 0.11a	​	0.66 ± 0.02b	3.52 ± 0.91a	MS, RI,S
*β*-Cedrene	546-28-1	1,445	1,432	-	7.21 ± 0.20a	-	0.48 ± 0.04b	MS, RI
(*E*)-*β*-Caryophylene	87-44-5	1,448	1,444	226.35 ± 10.29d	330.03 ± 10.24c	163.13 ± 1.58a	187.08 ± 2.96b	MS, RI, S
*α*-Humulen	6753-98-6	1,467	1,463	407.44 ± 3.94c	834.96 ± 5.86b	365.06 ± 0.53a	418.25 ± 2.92b	MS, RI, S
Alloaromadedrene	25246-27-9	1,471	1,467	11.19 ± 1.56c	24.54 ± 0.56a	11.09 ± 0.25d	14.82 ± 1.28b	MS, RI
*δ*-Cadinene	483-76-1	1,480	1,486	1.38 ± 0.16c	2.39 ± 0.04b	3.76 ± 0.77c	5.34 ± 0.80a	MS, RI, S
*Γ*-Cadinene	29350-73-0	1,483	1,480	28.08 ± 1.55c	68.09 ± 0.32a	34.34 ± 0.28b	49.19 ± 3.51c	MS, RI
*α*-Curcumene	644-30-4	1,487	1,486	1.46 ± 0.13b	3.84 ± 0.07a	1.09 ± 0.05c	3.38 ± 0.49a	MS, RI, S
*β*-Selinene	17066-67-0	1,501	1,500	4.49 ± 0.29a	10.6 ± 0.15a	6.68 ± 0.21d	8.46 ± 0.38b	MS, RI, S
Muurolene	10208-80-7	1,506	1,505	19.73 ± 0.57d	62.32 ± 1.21a	23.31 ± 0.21b	37.82 ± 0.48c	MS, RI
*β*-Bisabolene	495-61-4	1,512	1,512	13.42 ± 0.90c	51.22 ± 0.81a	23.02 ± 0.76d	44.53 ± 0.70b	MS, RI
*β*-Cadinene	29350-73-0	1,524	1,523	16.65 ± 1.13d	59.66 ± 0.24b	41.2 ± 0.48c	48.74 ± 0.13a	MS, RI
Calamenene	483-77-2	1,529	1,526	18.82 ± 1.70c	36.67 ± 0.7a	36.4 ± 1.46b	36.64 ± 1.05a	MS, RI
*α*-Calacorene	21391-99-1	1,553	1,550	0.98 ± 0.33a	-	-	0.65 ± 0.04a	MS, RI
Folenox	26619-69-2	1,561	-	0.38 ± 0.02a	-	-	-	MS, RI
Caryophyllene oxide	1139-30-6	1,593	1,593	187.1 ± 18.61b	289.27 ± 0.97a	58.47 ± 19.9c	232.42 ± 0.81a	MS, RI, S
Humulene oxide II	19888-34-7	1,620	1,620	146 ± 17.66d	287.25 ± 1.57b	96.73 ± 2.12c	188.61 ± 0.87a	MS, RI, S
***Terpenoids (26)***	​	​	​	**1343.02**	**2532.78**	**1036.44**	**1519.38**	​
Sulcatone	110-93-0	991	991	0.94 ± 0.27a	-	-	-	MS, RI, S
3,5-Octadienone	38284-27-4	1,079	1,076	0.44 ± 0.07a	-	-	-	MS, RI
Limona ketone	6090-09-1	1,144	1,144	0.22 ± 0.03a	-	-	-	MS, RI, S
(+)-2-Bornanone	464-49-3	1,166	1,161	0.55 ± 0.09a	-	-	-	MS, RI, S
Berbenone	80-57-9	1,225	1,223	0.21 ± 0.00a	-	-	-	MS, RI, S
*β*-Oploplenone	28305-60-4	1,577	1,573	0.56 ± 0.15b	-	-	1.25 ± 0.01a	MS, RI
cis-*β*-Elemenone	32663-57-3	1,603	1,597	2.55 ± 0.35b	1.31 ± 0.04c	1.19 ± 0.34d	4.52 ± 0.18a	MS, RI
***Ketones (7)***	​	​	​	**5.46**	**1.31**	**1.19**	**5.76**	​
Hendecane	1120-21-4	1,100	1,100	-	0.44 ± 0.01a	-	-	MS, RI, S
Dodecane	112-40-3	1,200	1,200	-	1.02 ± 0.01a	-	0.29 ± 0.07b	MS, RI, S
2,6-Dimethylundecane	17301-23-4	1,210	1,210	-	0.27 ± 0.01a	-	-	MS, RI, S
5-ethyl-5-methyldecane	17312-74-2	1,268	-	-	0.26 ± 0.01a	-	-	MS, RI
Tridecane	629-50-5	1,300	1,300	-	0.93 ± 0.03a	-	0.49 ± 0.12b	MS, RI, S
***Alkanes (5)***	​	​	​	**0.00**	**2.93**	**0.00**	**0.78**	​
1-Methylpyrrole	96-54-8	748	750	3.36 ± 1.44a	-	-	-	MS, RI, S
2-Pentylfuran	3777-69-3	995	994	-	0.70 ± 0.02a	-	-	MS, RI, S
***Heterocycles (2)***	​	​	​	**3.36**	**0.70**	**0.00**	**0.00**	​
Ethyl octoate	106-32-1	1,196	1,195	-	0.46 ± 0.01b	0.64 ± 0.00a	0.26 ± 0.04c	MS, RI, S
Ethyl pelargonate	123-29-5	1,295	1,294	-	1.18 ± 0.00b	0.74 ± 0.12b	0.51 ± 0.05a	MS, RI, S
***Esters (2)***	​	​	​	**0.00**	**1.64**	**1.38**	**0.77**	​

^a^ RI: Retention indices of compounds on DB-Wax column.

^b^ The lowercase letters (a-d) following the values in original sample and AOFEs, in the same row are significantly different according to Duncan’s test (*p* < 0.05).

^c^ Method of identification: MS, mass spectrum comparison using NIST20 library; RI, retention index in agreement with literature values; S, standard of volatiles.

^d^ Not detected.

The bold numbers provided in Table 3 represent the total value obtained by summing the contents.

The concentration of volatile compounds extracted by different processing methods also differed. The UAE -extracts had the highest total content of volatile compounds (2,638 μg/g), followed by the MAE-extracts (1,618 μg/g), and the HRE-extracts was the lowest (1,059 μg/g). After UAE processing, the concentration of almost all types of volatile compounds in AOF increased to varying degrees, especially for terpenoids. Terpenoids are key components that give AOF its distinctive aroma, and their high concentration reflects AOF’s inherent flavor characteristics (Zhang et al., 2007).

Terpenoids were a class of natural products composed of isoprene units that possess significant physiological and ecological functions, and were widely distributed in plants (Yang et al., 2020). Due to their low odor threshold and pleasant aroma, they significantly affected the aroma of AOF. The interactions between terpenoids themselves and with other types of components collectively form the unique flavor profile of AOFs. A total of 26 terpenoid components were identified, 18 of which were present in all four samples (Figure 4b). The content of terpenoids accounted for more than 95% of the total volatiles in AOF, mainly including sesquiterpenes (e.g., *α*-humulene, (*E*)-*β*-caryophylene, copaene) and oxygenated terpenes (e.g., (1*R*,7*S*)-germacra-4(15),5,10(14)-trien-1*β*-ol, humulene oxide II). Relevant studies had shown that volatiles such as *α*-humulene and humulene epoxide were the characteristic aroma components of AOF (Zhang et al., 2007). The content of these compounds was significantly affected by different processing methods. Compared with the original sample, the total terpenoid content in the UAE-extracts increased by 88.6% (from 1,343 μg/g to 2,533 μg/g), and the content of all detected terpenoid components increased to varying degrees. Notably, the content of *α*-humulene, which contributes to the aroma of AOF, increased to 835 μg/g; the content of humulene oxide II doubled; and the content of copaene increased to 307 μg/g. MAE-extracts maintained a moderate increase in total terpenoid content (1,519 μg/g) while significantly increasing the content of cadinene isomers. For HRE-extracts, high temperature caused significant degradation of terpenoids, resulting in severe loss of oxygenated terpenoid components (Vidic et al., 2018).

The main alcohols in AOFs were (1*R*,7*S*)-germacra-4(15),5,10(14)-trien-1*β*-ol (46 μg/g), contributing to the woody and sweet notes; spatulenol (5.29 μg/g), contributing to the herbal and fruity aroma; and viridflorol (2.62 μg/g), providing a sweet herbal note. Additionally, six trace components ((*Z*)-3-hexenol, borneol, 3-ethyl-4-methylpentanol, (-)-palustrol, (-)-cubenol, and (*S*)-verbenone) suffered significant loss during further processing. The total content of alcohols in UAE-extracts increased by 70%, reaching 96.4 μg/g, with elevated levels of (1*R*,7*S*)-germacra-4(15),5,10(14)-trien-1*β*-ol, spatulenol and viridflorol. These compounds enhanced the woody, herbal and sweet aromas of the extracts. However, although MAE processing increased the total volatile content in AOFs, the lower spatulenol content compared to UAE reduced the herbal and fruity notes in the sample. Prolonged HRE exposed AOFs alcohols to sustained heat conduction and residual oxygen, leading to hydrolysis and oxidative degradation of labile alcohols, and thus a significant loss of alcohol components (Lichtinger et al., 2024).

Except for terpenoids and alcohols, the volatile components of aldehydes, ketones, acids, and esters in AOFs underwent changes after processing. Following UAE treatment, the total content of aldehydes and acids remained largely unchanged, while the total ketone content decreased and ester content increased. MAE treatment resulted in a decrease in total aldehyde content, with little change in total acid and ketone content, while ester content increased but remained lower than in UAE and HRE treatments; HRE treatment led to an increase only in ester content, with decreases in the total content of all other components.

In summary, the aroma profile of AOFs were formed by the interactions among various compounds. Terpenoids and alcohols constituted the primary volatile flavor compounds, while ketones and aldehydes enriched the overall aroma profile of AOFs. Different processing methods significantly influenced the types and concentrations of volatiles in samples. Notably, UAE processing demonstrated markedly superior extraction efficiency for volatiles compared to MAE and HRE. This advantage likely stemmed from UAE’s enhanced ability to preserve the stability of volatiles during processing while leveraging cavitation effects to boost extraction rates of volatiles (Vinatoru et al., 2017). In contrast, microwave treatment caused the partial azeotropic vaporization of low-boiling-point terpenoids and alcohols in the presence of water. During condensation recovery, losses occurred due to shifts in vapor-liquid equilibrium, resulting in the characteristic aroma compounds of AOFs being extracted less efficiently by MAE than by UAE. Meanwhile, HRE involved prolonged high-temperature cooking, which leaded to the thermal transformation of certain compounds. High-boiling-point alcohols were enriched in the extracts via solvent reflux concentration. Consequently, the prepared extracts exhibited weaker aromatic harmony and quality than extracts obtained via UAE or MAE.

### 3.4 K-means cluster analysis of volatiles in AOFEs under different extraction methods

To more intuitively present the variation trends of volatile components in AOFs extracted by different processing methods, K-means clustering was applied to analyze the standardized concentrations of volatile. K-means clustering is a simple, intuitive and scalable unsupervised clustering algorithm. It partitions *n* data points into *k* clusters, each of which has a centroid. The algorithm then minimizes the distance between the internal data points and the centroid (Ikotun et al., 2023).

**FIGURE 5:**
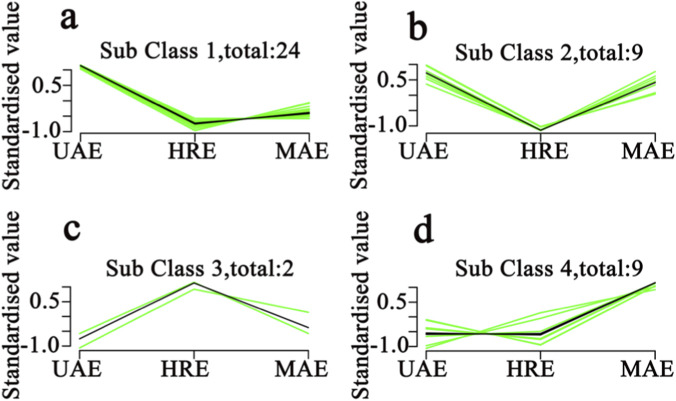
K-means results of volatile compounds in AOFEs with different processing methods (**(a–d)** represent Sub Class 1–4).

As shown in Figure 5, K-means clustering analysis could classify the volatile components of different AOFEs with the same variation trend into four subclasses. Subclasses one-four (Figures 5a–d) contained 24, nine, two, and nine volatile compounds, respectively. In Subclass one, the concentrations of 24 components in the UAE extracts were higher than in the other two samples. Most of these volatile components were terpenoids, such as *α*-copaene, *α*-humulene, *β*-caryophyllene and humulene epoxide. For the nine volatile components in subclass two, there was little difference in content between the UAE and MAE extracts, but both were higher than the HRE extracts. These components may include (1*R*,7*S*)-germacra-4(15),5,10(14)-trien-1*β*-ol, cis-*β*-copaene, caryophyllene, etc. The two components in Subclass three were found to have higher concentrations in the HRE sample, which were (-)-palustrol and ethyl octoate. The nine components in subclass four showed higher content in the MAE-extracts. Combined with Table 3, it could be seen that most of these compounds were only detected in the MAE-extracts, including *β*-oploplenone, 3-butylacrolein and decanal. In general, the contents of most compounds detected in the UAE sample were higher than those in the MAE and HRE samples, indicating that the extraction effect of UAE on the volatile components of AOF is superior to that of MAE and HRE.

### 3.5 Orthogonal partial least-squares discrimination analysis

**FIGURE 6:**
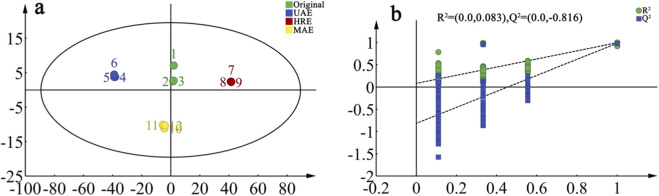
OPLS-DA score plots **(a)** and permutation test of volatile compounds **(b)** in AOF with different processing methods.

To investigate the effects of different processing methods on the volatile components of AOFs, the processing methods were employed as the independent variables and the 59 detected volatile components were taken as the dependent variables to conduct OPLS-DA, and the results are presented in Figure 6a. For this model, the independent variable fitting index (R^2^X) was 0.994, the dependent variable fitting index (R^2^Y) was 0.986, and the difference between R^2^X and R^2^Y was less than 0.3, which indicated that the model had high reliability and stability. The Q^2^ value was 0.968 > 0.5, suggesting that the model had strong predictive ability (Lv et al., 2025). A 200-time permutation test was conducted on the model (Figure 6b). It was found that the predictive index Q^2^ of the model intersected with the Y-axis in the negative semi-axis, with an intercept of −0.816. This indicated that the model had no overfitting and could be used to distinguish the volatile components of AOFEs by different processing methods. The original and HRE samples were in the first quadrant but distantly separated, the UAE sample in the second, and the MAE sample in the third, allowing clear differentiation of all four samples. Therefore, significant changes occurred in volatile compounds during processing, and the OPLS-DA model can effectively distinguish AOFEs processed by different methods.

The VIP value is used to evaluate the influence intensity and discriminative ability of each variable factor on the differences between sample groups. A higher VIP value indicates a greater difference in aroma components between groups and a more important role in classifying the aroma types of AOFs under different processing methods. Based on VIP > 1 and *P* < 0.05 (Table 4), 13 differential volatile components were screened out from the four samples, including 12 terpenoids and 1 alcohol.

**TABLE 4:** ROAV values of differential volatile components in AOFEs.

Compound	Aroma description^a^	Threshold (μg/g)^b^	ROAV			*P*	VIP
			UAE	HRE	MAE		
Cubebene	Herbal	-	-	-	-	**	1.03
(1*R*,7*S*)-Germacra-4(15),5,10(14)-trien-1*β*-ol	Sweet, woody	1.80	1.79	0.83	3.44	**	1.63
Muurolene	Woody	-	-	-	-	**	1.28
Copaene	Woody, sweet	6.00	2.39	1.88	1.93	**	2.43
*α*-Humulen	Woody, floral	0.39	100.00	91.81	91.72	**	3.66
*β*-Cadinene	Spicy, woody	1.50	1.86	2.69	2.78	**	1.54
*β*-Bisabolene	Woody	-	-	-	-	**	1.51
(*E*)-*β*-Caryophylene	Sweet, woody, floral	0.16	96.35	100.00	100.00	**	2.61
*Γ*-Cadinene	Woody, herbal	1.50	2.12	2.24	2.81	**	1.21
Humulene oxide II	-	0.45	29.82	21.08	35.85	**	2.15
Calamenene	Sweet, woody, herbal	-	-	-	-	**	1.11
cis-*β*-Copaene	Woody, herbal	-	-	-	-	**	1.12
Caryophyllene oxide	Sweet, woody	5.50	2.46	1.04	3.61	**	2.61

^a^ Aroma description was obtained from http://www.thegoodscentscompany.com.

^b^ Threshold (in water) was quoted from the literature data (Van Gemert, 2011).

**FIGURE 7:**
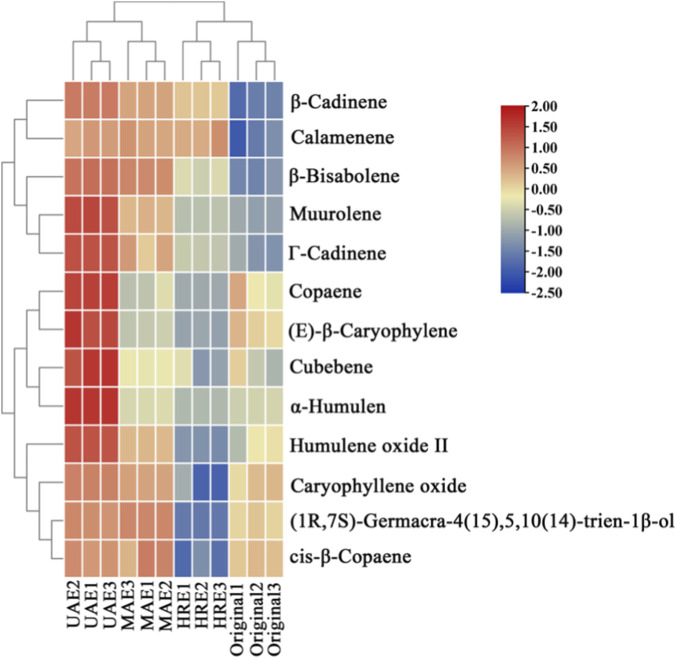
Cluster heatmap of differential volatile components in AOF with different possessing methods.

To more intuitively present the variation pattern of volatile component contents in AOF under different processing methods, heatmap and HCA were performed on the 13 screened differential compounds, and the results were shown in Figure 7. All samples were clearly divided by HCA, and the contents of the 13 differential volatile components in the UAE-extracts were all higher than those in the original sample; in particular, the contents of characteristic aroma components of AOFs (such as copaene, (*E*)-*β*-caryophylene, *α*-humulene, and humulene oxide II) were much higher than those in the original sample. This indicated that UAE can effectively extract the key volatile components of AOFs.

MAE and HRE extracts showed higher concentrations of *β*-cadinene, calamenene, *β*-bisabolene, muurolene and *Γ*-cadinene than the original sample. However, HRE-extracts had much lower caryophyllene oxide and humulene oxide II levels than the original, while MAE had similar copaene, (*E*)-*β*-caryophylene, *α*-humulene and humulene oxide II concentrations to the original. The results suggested that, while heat-assisted extraction can extract the volatile components of AOFs, exposure to a long-term high-temperature environment can cause significant degradation of terpenoids, especially oxygenated terpenoids such as humulene epoxide and caryophyllene, resulting in severe loss. The result obtained was consistent with the results of aroma types and contents in Section 3.3, which further verified the reliability of the OPLS-DA model.

### 3.6 Odor profiles of AOFEs

In the assessment of key flavor compounds in food, the concentration of volatile components does not directly determine their contribution to aroma profile. A comprehensive analysis must therefore incorporate the odor thresholds of each compound. ROAV is an evaluation index used to objectively identify key flavor compounds based on odor thresholds (Li et al., 2024). In the current study, ROAVs of 13 compounds (VIP > 1 and *p* < 0.05) were calculated by combining the relative contents of differential volatiles and their odor thresholds. *α*-Humulene exhibited a relatively high content in UAE-extracts with a threshold of 0.39 μg/g, contributing most significantly to the overall aroma profile of UAE-extracts. Therefore, its ROAV was defined as 100. Similarly, the ROAV of (*E*)-*β*-caryophylene was defined as 100 in both HRE and MAE extracts. The results (Table 4) showed that there were eight differential volatile components with ROAV > 1. All eight differential volatiles in the UAE and MAE extracts had ROAV > 1. In HRE-extracts, seven components had ROAV > 1, with the exception of (1*R*,7*S*)-germacra-4(15),5,10(14)-trien-1*β*-ol, which imparts a woody and sweet aroma. The common differential volatile components with ROAV > 1 in three extracts were copaene, *α*-humulene, *β*-cadinene, (*E*)-*β*-caryophylene, *Γ*-cadinene, humulene oxide II, and caryophyllene oxide. These compounds were all terpenoids, mostly with sweet, floral, and woody notes, making significant contributions to the overall aroma of the three extracts and thus can be identified as key differential aroma compounds in AOFEs under different processing methods. (1*R*,7*S*)-germacra-4(15),5,10(14)-trien-1*β*-ol was a key differential aroma compound in MAE and UAE extracts but only played a modifying role in the overall aroma of the HRE extracts. Additionally, there were no reported thresholds for the other five volatile compounds, and the corresponding reference standards were not commercially available. However, based on their aroma attributes and concentrations, these five compounds exert a certain effect on the overall aroma of AOFEs.

**FIGURE 8:**
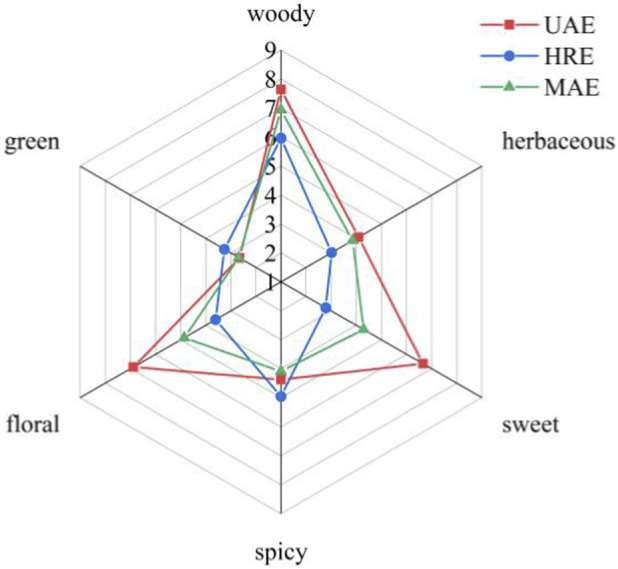
Sensory evaluation of AOFEs with different possessing methods.

Sensory evaluation plays a crucial role in assessing consumers’ preference for spice products. A descriptive sensory analysis of AOFEs was conducted, and the results were showed by the radar diagram in Figure 8. Overall, the most prominent aroma in all three extracts was woody. While different extraction methods had a significant impact on the aroma attributes of the extracts, with the UAE-extracts has floral, sweet, and woody notes, with the woody note being the strongest, and the MAE extracts having a similar score in woody note to the UAE extract, but with their spicy, floral, and sweet notes much weaker than those of the UAE extracts. Compared with the UAE and MAE extracts, the HRE extracts had more pronounced green and spicy notes. Sensory evaluation indicated that processing methods could significantly affect the aroma characteristics of AOFEs. The UAE improved mass transfer efficiency by utilizing the cavitation effect, which achieved a diverse and complex extraction effect on the volatile compounds of AOF (Zhang et al., 2025).

### 3.7 Correlation analysis

**FIGURE 9:**
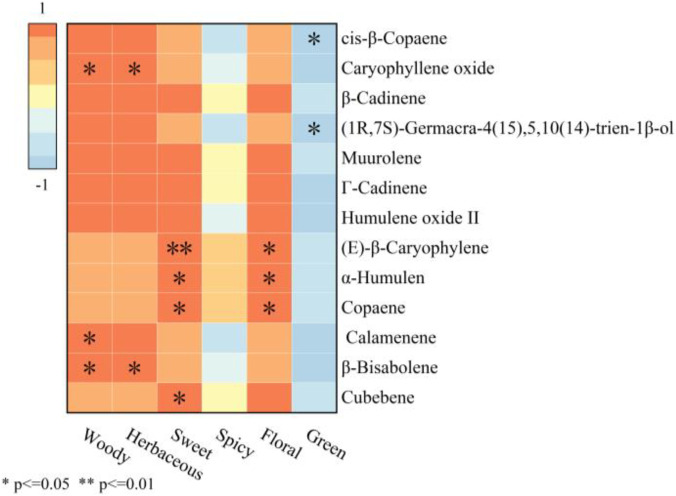
Pearson correlation analysis between sensory evaluation and differential aroma compounds.

To clarify the influence of differential components on the aroma attributes of AOFEs under different extraction methods, Pearson correlation analysis was conducted between the 13 screened differential aroma components and the sensory aroma attributes of AOFEs, with the results shown in Figure 9. The woody, herbal, sweet and floral notes of AOFEs were positively correlated with all 13 differential aroma components, whereas the green note was negatively correlated. Specifically, caryophyllene oxide, calamenene, and *β*-bisabolene were positively correlated with the woody note. The herbal note showed a significant positive correlation with caryophyllene oxide and *β*-bisabolene. The sweet note showed an extremely significant positive correlation with (*E*)-*β*-caryophylene and a significant positive correlation with *α*-humulene, copaene, and cubebene. The floral note showed a significant positive correlation with (*E*)-*β*-caryophylene, *α*-humulene, and copaene. The green and spicy notes were negatively correlated with most of the screened differential aroma components. The green note was significantly negatively correlated with cis-*β*-copaene and (1*R*,7*S*)-germacra-4(15),5,10(14)-trien-1*β*-ol, while cis-*β*-copaene, caryophyllene oxide, and *β*-bisabolene were negatively correlated with the spicy note. The 13 screened differential aroma components exhibited synergistic or modifying effects with each other, collectively shaping the unique aroma profile of the AOFEs.

## 4 Conclusion

This study employed HS-SPME to evaluate the extraction efficiency of different fiber-coated probes for volatile components in AOFs. A SPME probe coated with DVB/CAR/PDMS was selected for GC-MS analysis of volatile components in AOFEs prepared via UAE, MAE, and HRE methods and original samples. A total of 59 volatile components were detected, and UAE extracts had the highest number and content of terpenoid components, which was basically consistent with the test results of the E-nose. K-means clustering analysis classified the volatile components of the AOFEs prepared by the three methods into four subclasses based on their content variation trends. The contents of most compounds in the UAE-extracts were higher than those in the MAE and HRE extracts. OPLS-DA combined with VIP values screened out 13 compounds as the differential volatiles for distinguishing the AOFEs prepared by the three processing methods. Further ROAV analysis of these differential volatile components confirmed that (1*R*,7*S*)-germacra-4(15),5,10(14)-trien-1*β*-ol, copaene, *α*-humulene, *β*-cadinene, (*E*)-*β*-caryophylene, *Γ*-cadinene, humulene oxide II, and caryophyllene oxide were the key differential volatile components of the AOFEs under the three processing methods. Pearson correlation analysis showed that the sweet, floral, herbal, and woody notes of the extracts were positively correlated with most differential volatile compounds, while the green and spicy notes were negatively correlated with most differential volatile compounds. The findings of this study could provide scientific validation for the development of AOFEs and could serve as a reference point for the chemical analysis of the aromatic properties of other AOF-derived products.

## Data Availability

The raw data supporting the conclusions of this article will be made available by the authors, without undue reservation.
